# Ehrlichioses: An Important One Health Opportunity

**DOI:** 10.3390/vetsci3030020

**Published:** 2016-08-31

**Authors:** Tais B. Saito, David H. Walker

**Affiliations:** Department of Pathology, University of Texas Medical Branch at Galveston, Galveston, TX 77555, USA; dwalker@utmb.edu

**Keywords:** *Ehrlichia canis*, *Ehrlichia chaffeensis*, *Ehrlichia ewingii*, *Ehrlichia muris*, *Ehrlichia ruminantium*, canine ehrlichiosis, human ehrlichiosis, heartwater, immunopathology, mouse models

## Abstract

Ehrlichioses are caused by obligately intracellular bacteria that are maintained subclinically in a persistently infected vertebrate host and a tick vector. The most severe life-threatening illnesses, such as human monocytotropic ehrlichiosis and heartwater, occur in incidental hosts. *Ehrlichia* have a developmental cycle involving an infectious, nonreplicating, dense core cell and a noninfectious, replicating reticulate cell. Ehrlichiae secrete proteins that bind to host cytoplasmic proteins and nuclear chromatin, manipulating the host cell environment to their advantage. Severe disease in immunocompetent hosts is mediated in large part by immunologic and inflammatory mechanisms, including overproduction of tumor necrosis factor α (TNF-α), which is produced by CD8 T lymphocytes, and interleukin-10 (IL-10). Immune components that contribute to control of ehrlichial infection include CD4 and CD8 T cells, natural killer (NK) cells, interferon-γ (IFN-γ), IL-12, and antibodies. Some immune components, such as TNF-α, perforin, and CD8 T cells, play both pathogenic and protective roles. In contrast with the immunocompetent host, which may die with few detectable organisms owing to the overly strong immune response, immunodeficient hosts die with overwhelming infection and large quantities of organisms in the tissues. Vaccine development is challenging because of antigenic diversity of *E. ruminantium*, the necessity of avoiding an immunopathologic response, and incomplete knowledge of the protective antigens.

## 1. Introduction

Ehrlichiae are obligately intracellular bacteria that are maintained in natural cycles involving a persistently infected vertebrate host and a tick vector. In addition to wildlife reservoir hosts—such as white-tailed deer, African ruminants, carnivores, and rodents—there are incidental hosts: domestic livestock, companion animals, and humans. Infections appear to be subclinical in natural hosts, and the most severe life-threatening illnesses, such as human monocytotropic ehrlichiosis and heartwater, occur in incidental hosts. Understanding the biology of persistent subclinical infection and severe disease manifestations is a worthy goal of both veterinary and human medical scientists. Knowledge is being pursued by scientists in both veterinary and human medicine of the immunological mechanisms of clearance of the pathogens and resolution of the pathologic lesions, the host and ehrlichial pathogenic mechanisms mediating persistent infection, and the pathogenic mechanisms of the severe disease. 

## 2. History of Ehrlichioses and Anaplasmoses

Ehrlichioses were the sole province of veterinary medicine until a case report of a man infected with an agent that was identified as an *Ehrlichia* by electron microscopy of circulating monocytes containing intravacuolar bacteria with the characteristic ultrastructural appearance of *Ehrlichia* accompanied by the development of antibodies reactive with *Ehrlichia canis* [1]. The actual pathogen, *E. chaffeensis*, was cultivated in 1991 and demonstrated to possess antigens shared with *E. canis* [2].

The history of veterinary discoveries of ehrlichioses long preceded these events. Theiler [3] identified a related pathogen in the family Anaplasmataceae, *Anaplasma marginale*, in 1910. Cowdry [4] identified the etiologic agent of heartwater in 1925, and Donatien and Lestoquard [5] determined that *E. canis* is the causative pathogen of canine monocytic ehrlichiosis in 1935. All of these discoveries were made in Africa. In 1940 Gordon et al. [6] identified *Anaplasma phagocytophilum* as the agent of tick-borne fever in the United Kingdom, and Ewing et al. [7] identified the agent of canine granulocytic ehrlichiosis that now bears his name, *E.* *ewingii*, in Oklahoma in 1971. These organisms were known through the years by several different names until molecular phylogeny sorted out their true genetic relationships in 1999 [8].

Additional human ehrlichioses have subsequently been discovered. In a private practice, infectious diseases subspecialist in Duluth, Minnesota, Johan Bakken, observed a disease in several of his patients who had cytoplasmic inclusions in some of their circulating neutrophils [9]. He suspected that these were ehrlichiae on the basis of their similarity to a photomicrograph in an update to the ehrlichiosis chapter in Mandell’s textbook on infectious diseases that had recently arrived in the mail. Because Walker was the author of the update, Bakken sent blood smears, sera, and blood from patients, and paraffin blocks from an autopsy to his laboratory at the University of Texas Medical Branch at Galveston where J. Stephen Dumler led their study. After exhaustively determining that the patients did not have *E. chaffeensis* infection, a novel technique developed by Relman [10] was applied to DNA extracted from the patients’ blood, namely polymerase chain reaction with universal primers for the eubacterial *rrs* gene. Laborious manual sequencing by Chen et al. [11] identified the agent as what was then known as *E. phagocytophila* [11]. Dumler was continuously involved in the project after completion of his fellowship and returning to the School of Medicine of the University of Maryland as a member of the faculty, contributing serological data and ultrastructural detection of the agent in autopsy tissue. The organism was cultivated by Goodman et al. [12] in 1996 and renamed *A.* *phagocytophilum* by Dumler et al. [8] in 1999. Subsequently, two additional human ehrlichioses and one additional human anaplasmosis have been discovered. Human infections with *E. ewingii* were identified in Missouri in 1999, and *E. muris* infections of patients in Minnesota and Wisconsin were discovered by Pritt et al. [13] in 2009 [14]. Human infections with a novel *Anaplasma*, *A. capra*, were discovered in China in 2015 [15].

## 3. The Agents of Ehrlichioses

Among the five genera in the family Anaplasmataceae of the order Rickettsiales, namely *Anaplasma*, *Ehrlichia*, *Neorickettsia*, *Neoehrlichia*, and *Wolbachia*, this article focuses on the diseases caused by organisms of the genus *Ehrlichia*.

Ehrlichiae undergo a developmental cycle with two physiologic forms: an infectious, nonreplicating, smaller (0.4–0.6 µm) dense-core form with ribosomes and DNA condensed in the center of the bacterium, and a larger (0.4–0.6 µm × 0.7–1.9 µm) noninfectious reticulate cell form that replicates by binary fission in a cytoplasmic vacuole [16,17,18]. The cytoplasmic vacuole containing a microcolony of ehrlichiae is the structure that is detected in circulating leukocytes by Romanovsky staining and has been called a *morula* (Latin for mulberry) because of its resemblance to that fruit. The cell wall of *Ehrlichia* lacks the structural components that are immunological pattern recognition molecules, lipopolysaccharide and peptidoglycan, a hint of the ehrlichial stealth infection strategy. The ehrlichial cell wall contains several proteins that have tandem repeat units which perform various functions, including adhesion to the host cell membrane, binding of host cytoplasmic proteins after secretion by a type I secretion system, and translocation to the host cell nucleus where they bind to DNA to stimulate and inhibit transcription of host cell genes with effects that favor ehrlichial survival. Ehrlichiae also have a 200 kDa protein, which resembles host cell ankyrin, that is also translocated to the host cell nucleus and binds host cell chromatin. In addition, the ehrlichial cell wall also contains one or more of the family of 28 kDa proteins that are encoded by a locus in the circular 1.2–1.5 × 10^6^ bp genome [19,20,21,22]. The 28 kDa protein that is expressed appears to be related to adaptation to different hosts, e.g., p28-19 of *E*. *chaffeensis* is expressed in mammalian hosts, and p28-14 is expressed in the tick host [23]. The p28 locus in different species of *Ehrlichia* varies in size and number of p28 genes, often more than 20 genes. The 22 p28 kDa proteins of *E. chaffeensis* contain three hydrophilic, surface-exposed hypervariable domains. The small genomes, a result of reductive evolution, contain more than 400 conserved housekeeping genes and more than 300 hypothetical genes whose functions are unknown but likely are critical for the obligately intracellular lifestyle.

## 4. Clinical Manifestations

### 4.1. Human Ehrlichioses

Initially, human monocytotropic ehrlichiosis presents as an undifferentiated febrile illness with fever, headache, myalgia, and malaise [24]. Manifestations of multisystem disease such as nausea, vomiting, diarrhea, abdominal tenderness, regional lymphadenopathy, cough, rash, stiff neck, photophobia, and confusion occur in 20%–40% of patients. Meningoencephalitis occurs in 20%, and acute respiratory distress syndrome occurs in the most severe cases. Hospitalization is required in 40%–60% of cases, and 2% of cases are fatal [24,25]. Characteristic laboratory abnormalities are leukopenia with both lymphocytopenia and neutropenia, thrombocytopenia, and elevated serum concentrations of hepatic transaminases. Pathologic lesions include meningoencephalitis, interstitial pneumonia, multifocal hepatocellular death, bone marrow containing granulomas likely reflecting clearance of the bacteria, erythrophagocytosis, myeloid hyperplasia, and megakaryocytosis, likely reflecting the mechanism of cytopenias and the compensatory responses to the cytopenias [26]. In immunocompetent persons even severe illness is associated with a paucity of organisms in the tissues, suggesting immunopathogenic mechanisms. In contrast, human monocytotropic ehrlichiosis in immunodeficient patients, such as persons with human immunodeficiency virus associated acquired immunodeficiency syndrome, is a severe illness with overwhelming quantities of ehrlichiae in the blood and organs [27].

Human infections with *E*. *ewingii* and *E. muris* have similar, although less severe, clinical presentations as human monocytotropic ehrlichiosis. No fatalities have been reported with either of these human ehrlichioses [14,27].

Febrile human illnesses associated with detection of antibodies reactive with *E. chaffeensis* have been observed in patients and the Perm region of Russia, where DNA sequences identical to those of *E. muris* were detected in *Ixodes persulcatus* ticks. This situation suggests the potential for a wider global distribution of human infections with *E. muris* [28].

### 4.2. Canine Monocytic Ehrlichiosis

*Ehrlichia canis*, *E. chaffeensis*, and *E. ewingii* infect dogs, but also have been reported to cause infection in humans. Canine monocytic ehrlichiosis caused by *E. canis* induces clinical and hematological changes that can be serious and even fatal. Vertebrate reservoirs of *E. canis* are limited to the family Canidae. *Ehrlichia canis* is transmitted by the brown dog tick, *Rhipicephalus sanguineus*, and the bacteria are maintained through the tick life stages by transstadial, but not by transovarian, transmission [8,29]. In the tick, the microorganism spreads from the midgut to the hemocele and in hemocytes to the salivary gland, and infection of the host occurs through the tick’s salivary secretions in the site of the vector feeding.

Usually there is a history of tick infestation that is associated with nonspecific clinical manifestations [30,31]. The course of the disease classically can be divided into three phases: acute, subclinical, and chronic. The acute phase occurs after an incubation period of 8–20 days and persists for 2–4 weeks, during which there is multiplication of the bacteria in mononuclear cells, and spread of ehrlichiae within the host [32,33]. Canine ehrlichiosis may occur as a multisystemic disease, with or without bleeding, such as petechiae and/or dermal ecchymosis. Lymphadenopathy and/or splenomegaly occurs in 20%–25% of cases. Ocular signs may include anterior uveitis, chorioretinitis, papilledema, retinal hemorrhage, perivascular inflammation, retinal detachment, and even blindness. Convulsions, stupor, ataxia, central or peripheral vestibular dysfunction, cerebellar dysfunction, anisocoria, tremor, and generalized or localized hyperesthesia are observed in some infected animals as a result of meningitis or cerebral hemorrhage. Dogs with ehrlichial infection can also present with hemarthrosis or deposition of immune complexes in joints [31,34,35,36]. Hematologic changes are associated with autoimmunity or coagulopathy, including anti-platelet antibodies, persistent thrombocytopenia with increased numbers of large regenerative platelets, variable mild leukopenia, non-regenerative anemia, and hemolysis. The acute clinical phase resolves spontaneously [30,31,32,33,37]. Immunocompetent animals can eliminate the agent, even without specific treatment, or remain persistently infected reservoirs of the pathogen [37,38]. These animals enter the subclinical phase during which they appear healthy. Subsequently, these animals may develop pancytopenia associated with hypoplastic bone marrow failure leading to death.

### 4.3. Canine Granulocytic Ehrlichiosis

The primary vector of *E. ewingii* is *A. americanum*, the lone star tick [39]. The natural host is white-tailed deer, which can harbor infectious organisms, and the dog is another possible reservoir [40,41,42]. Dogs inoculated experimentally with *E. ewingii* develop fever, thrombocytopenia, and leukopenia. Bacteria are detected in blood around 10 days post infection, morulae are detected in neutrophils around 20 days post infection, and seroconversion occurs between 7 and 21 days, depending on the route of infection [43]. Dogs infected via tick-transmission develop detectable *E. ewingii* DNA in the blood between days 11 and 28. The infection dynamics of *E. ewingii* in dogs varies greatly based on the dose of inoculum and previous infection status. Dogs previously infected with *E. ewingii* may develop active infection upon challenge. Unlike dogs infected with *E. ewingii* for the first time, they do not develop fever or thrombocytopenia. Intermittent fever is common only among dogs during a primary infection with *E. ewingii* and in naturally infected dogs [44,45]. Less common presentations such as lameness, neutrophilic polyarthritis, anemia, peripheral edema, and lymphadenopathy may also occur in naturally infected dogs. Numerous hematologic and biochemical abnormalities have been associated with natural infections of *E. ewingii* in dogs and humans, including thrombocytopenia, leukopenia, high serum alkaline phosphatase concentration, high serum alanine aminotransferase, hypokalemia, hyperglobulinemia, hyperphosphatemia, hypophosphatemia, and hypocalcemia [14,44,45]. Thrombocytopenia may persist for several weeks.

### 4.4. Heartwater

The clinical disease caused by *Ehrlichia* (formerly *Cowdria*) *ruminantium* affects domestic and wild ruminants in many African countries and on Caribbean islands, and often causes death of clinically affected ruminants [46]. 

Infected animals present a peracute, acute, subacute, or subclinical form. The peracute form is relatively rare. High fever, respiratory distress, terminal convulsions, and diarrhea may precede death during the peracute form. The acute form is most often observed and is characterized by fever, inappetence, depression, listlessness, moist cough, bronchial rales, cyanosis of mucous membranes, dyspnea, and CNS signs. The acute form of disease is typically fatal in less than 1 week from the onset of clinical signs. The subacute form of heartwater, which is rarely encountered, is characterized by prolonged fever, cough, and mild incoordination. Affected animals recover or die within 1–2 weeks after the onset of the subacute form of disease. A mild or subclinical form of heartwater is characterized by transient fever and may occur in partially immune cattle or sheep, or calves that are younger than 3 weeks old [47].

### 4.5. Coinfections of Ehrlichia and Other Tick-Borne Organisms

Coinfections of ehrlichiae and other tick-borne agents have rarely been reported. A patient in North Carolina developed fever, headache, myalgia, nonproductive cough, nausea, and vomiting followed by worsening symptoms and a rash three days later. Laboratory studies revealed leukopenia, thrombocytopenia, hyponatremia, and mildly elevated hepatic transaminases. He reported tick bite and crushing ticks removed from his dog. Immunofluorescence staining of a skin lesion identified spotted fever group rickettsiae. Immunofluorescence assay (IFA) antibody titers to *E. chaffeensis* increased four-fold, from 32 to 128 [48].

Because the most highly prevalent tick in North Carolina is *Amblyomma americanum*, which is host for *R. amblyommii, R. parkeri*, and *E. chaffeensis*, it is most likely that the patient was coinfected with *E. chaffeensis* and either *R. amblyommii* or *R. parkeri.* Half of the *A. americanum* ticks in North Carolina carry *R. amblyommii*, and indirect evidence strongly suggests that this species can cause subclinical infection and self-limited illness. Although *R. parkeri* is found in a high proportion of *A. maculatum* ticks, its presence in a smaller portion of the highly prevalent *A. americanum* ticks may also result in human infections.

Studies at Fort Chaffee, Arkansas revealed that a substantial portion of soldiers, during summer exercises seroconverted to spotted fever group rickettsiae. The majority of these seroconversions were asymptomatic. However, at an odds ratio greater than 2, soldiers that seroconverted had a greater incidence of headache, myalgia, rash, joint pain, fever, chills, dyspnea, and confusion than nonseroconverters [49]. The ticks to which the soldiers were exposed were nearly all *A. americanum*, a very high proportion of which carry *R. amblyommii* in Arkansas. Experimental infections of guinea pigs with *R. amblyommii* cause a high titer seroconversion without illness [50].

*Ixodes scapularis*, the tick vector of *E. muris*, also transmits *Anaplasma phagocytophilum, Babesia microti, Borrelia burgdorferi*, and Powassan virus. Although coinfections of *E. muris* and these other agents have not been reported, there is a possibility of their occurrence. Coinfection does not have to result from transmission by a single dually infected tick because tick-exposed persons may experience more than one tick bite.

## 5. Pathogenesis of Host-*Ehrlichia* Interactions

### 5.1. Human Monocytotropic Ehrlichiosis

Apparently the vertebrate host-*Ehrlichia* interaction for a particular species of *Ehrlichia* is determined mainly by the host. For example, *E. chaffeensis* does not seem to cause significant disease in its natural host, although variable fever on days 17–21 post-inoculation has been observed in experimentally infected deer [51]. Paradoxically, severe disease occurs in some patients in association with a strong, possibly excessive, immune response, and fatalities also occur in severely immunocompromised persons. Severe disease in immunocompetent patients is associated with a paucity of organisms in the tissues, presumably as a result of an excessive immune response. On the other hand, severely immunocompromised patients’ tissues contain large quantities of ehrlichiae.

A study comparing the host responses of a patient who suffered fatal human monocytotropic ehrlichiosis and a patient with mild *E. chaffeensis* infection revealed results similar to those of animal models of ehrlichiosis [52]. The fatal case was characterized by anemia, thrombocytopenia, and severe leukopenia and lymphopenia, increased serum hepatic transaminases, respiratory distress with hypoxia, seizure-like activity, multiorgan failure, and septic shock. The mild case had a three-day history of fever, myalgias, and headache that resolved with doxycycline therapy. The fatal case was associated with dysregulated cytokine and chemokine production with markedly higher concentrations of proinflammatory cytokines (tumor necrosis factor α (TNF-α), interferon-γ (IFN-γ), interleukin-1α (IL-1α), IL-1β, and IL-6) and chemokines (monocyte chemoattractant protein-1 (MCP-1), macrophage inflammatory protein-1α (MIP-1α), and MIP-1β) that may have contributed to the multiorgan failure. The fatal case also had markedly increased levels of anti-inflammatory cytokines (IL-10 and IL-13) that may have caused the weak T helper 1 (Th1) cell-mediated immunity (low observed concentrations of IFN-γ and IL-2 and severe lymphopenia). The fatal disease was also associated with greatly increased IL-8 and granulocyte-colony stimulating factor (G-CSF), possibly compensatory responses to the neutropenia.

### 5.2. Canine Monocytic Ehrlichiosis

After 7–12 days of *E. canis* infection ehrlichial morulae are detected in peripheral blood. Mononuclear cells in lymphoid organs release the dense-core ehrlichiae by exocytosis or lysis to infect other cells. Multiplication occurs primarily in macrophages and lymphocytes, resulting in lymphoreticular hyperplasia, and may result in enlargement of spleen, liver, and lymph nodes. Infected cells interact with the endothelial cells of the smaller blood vessels, causing vasculitis, and perivascular migration of macrophages and lymphocytes [53]. 

An antibody response is observed 13 days after infection [30,54,55], with a peak at 56–70 days [55,56]. Humoral immunity by itself is insufficient to extinguish infection by *E. canis* [57]. Most infected dogs survive the acute phase. Animals treated with antibiotics or those untreated may evolve into an asymptomatic phase and maintain a persistent infection [58,59,60]. 

In canine ehrlichiosis, the persistence of the ehrlichial agent in the face of high titers of antibodies [37,61] demonstrate that, for effective clearance of the microorganism, the cellular immune response of the host is required [62]. The maintenance of high titers of circulating antibodies against *E. canis* reflects persistent antigenic stimulation [37,58]. Another feature of the humoral response in ehrlichial infections is plasmocytosis in most affected organs, including bone marrow [63,64]. 

Genetic susceptibility or resistance of different canine breeds and the mode of transmission can affect the course of the infection. Some studies suggest that the disease is mediated by the immune response more than by the direct effects of the bacteria [65,66,67].

The concentrations of total proteins, total globulin, α-2 globulin, β-2 globulin and γ-globulin are substantially elevated in infected dogs, and the albumin/globulin ratio is low in infected dogs. Polyclonal gammopathy is reflected in hypergammaglobulinemia [66,68]. 

Neutropenia in infected dogs suggests that these animals are immunocompromised and, therefore, more susceptible to secondary infections that can result in an unfavorable outcome [68]. The high anti-*E. canis* antibody concentration detected in asymptomatic dogs for months after the acute phase of infection, as well as an absolute lymphocytosis and polyclonal gammopathy, indicate a prolonged duration of infection and chronic antigenic stimulation, reflecting the inability of some animals to naturally eliminate the infection after the acute phase of the disease [37,69,70]. 

Thrombocytopenia and thrombocytopathy observed in ehrlichiosis involve immunological and inflammatory mechanisms related to increased consumption and decreased half-life of platelets, likely due to splenic sequestration. IgG antibodies directed against platelet proteins can lead to reduction in the number of platelets. The serum level of a particular cytokine, platelet migration inhibition factor, is inversely proportional to the platelet count with elevations associated with more virulent strains. 

Ehrlichiae targeting the innate immune system activate the production of IL-12 by antigen-presenting cells, inducing the development of a Th1 response and IFN-γ secretion by CD4 T cells, which plays a role in host control of ehrlichial infection. Lymphoid organs such as spleen and lymph nodes of dogs experimentally infected with *E. canis* contain cells with reduced expression of molecules of major histocompatibility complex class II (MHC II) [63]. In ehrlichial infections, there is an inverted CD4:CD8 ratio of T lymphocytes in peripheral blood; more specifically, there is a reduction in levels of CD4+ T cells [55,71]. 

Investigation of the expression of mRNA of cytokines in dogs experimentally infected by *E. canis* demonstrates persistent expression of TNF-α and IFN-γ mRNA, and low levels of mRNA of IL-1β, IL-2, IL-4, and IL-6 throughout 56 days of infection [72]. In clinically moderate ehrlichiosis there is persistent elevation of IL-1β and IL-8, and in dogs with severe acute ehrlichiosis there are reduced levels of IFN-γ and TNF-α [73].

In splenectomized dogs, the clinical and hematological changes are mild compared to intact animals. Significant differences observed between splenectomized and intact dogs can be explained by the mechanisms involved in the pathogenesis of canine ehrlichiosis involving erythrophagocytosis, leukophagocytosis, and thrombocytophagocytosis [74]. The spleen is probably the last organ to maintain *E. canis* infection during recovery, possibly in splenic macrophages, avoiding immune clearance [37].

### 5.3. Canine Granulocytic Ehrlichiosis

Very little is known about *E. ewingii* and the pathogenesis of canine granulocytic ehrlichiosis. Neutrophils taken from a dog naturally infected with *E. ewingii* have been maintained for various periods in cell culture, but continuous productive replication has not been achieved. The percentage of apoptotic neutrophils in infected dogs is significantly lower than in uninfected dogs after culture, suggesting that neutrophil apoptosis is delayed by *E. ewingii* infection allowing further ehrlichial growth. *Ehrlichia ewingii* infection delays the loss of mitochondrial membrane potential associated with spontaneous apoptosis in neutrophils [75].

### 5.4. Heartwater

Once inoculated into the host, *E. ruminantium* typically resides inside intracytoplasmic inclusions in neutrophils and endothelial cells [76,77]. Different strains of *E. ruminantium* often fail to induce heterologous cross-protection, apparently because of antigenic diversity [76]. Genetic diversity also impacts the degree of pathogenicity of various strains of *E. ruminantium*. Some strains are highly virulent; others appear to be nonpathogenic [78,79].

*Amblyomma* spp. ticks transmit *E. ruminantium* to susceptible ruminant hosts. In Africa, there are five native tick species, *A. variegatum*, *A. hebraeum*, *A. lepidum*, *A. astrion*, and *A. pomposum* that are considered natural vectors of heartwater. Introduction of cattle infested with *A. variegatum* led to the presence of heartwater on Caribbean islands. Domestic cattle, sheep, and goats are target species for heartwater, as well as other less familiar domestic ruminants. 

The tick cycle of the ehrlichial infection begins in the intestinal epithelial cells and spreads via the hemolymph to the tick salivary glands. *E. ruminantium* can be transmitted transstadially, but rarely, if ever, transovarially [80,81]. Only a portion of infected ticks transmit *E. ruminantium* during feeding, and transmission requires 2–3 days (38 h) for nymphs and up to 4 days for adult ticks [80,81].

*E. ruminantium* organisms delivered by infective tick challenge are more virulent than organisms delivered as needle challenge with infected blood [82,83].

Vertical transmission occurs from cows to neonatal calves by ingestion of *E. ruminantium*-infected leukocytes in colostrum [84]. Subcutaneous, intramuscular, and intraperitoneal inoculation of infective blood into susceptible animals can also initiate infection [80].

Initial replication takes place in cells of the mononuclear phagocytic system in the regional lymph nodes, and dissemination occurs via the blood stream with invasion of vascular endothelial cells. Antibodies do not seem to control the course of the disease. 

After transmission the organisms reside in regional lymph nodes where replication occurs [85]. The febrile stage develops between 3 and 10 days after transmission, at which time organisms are located in plasma cells, neutrophils and, occasionally, other granulocytes [86,87]. 

Genetic factors are important for the development of immunity against heartwater. Immunity is probably maintained best in sheep; in contrast, immunity is of the shortest duration in cattle [86]. Calves, kids, and lambs have natural resistance against heartwater during the neonatal period, and resistance appears to be unrelated to the immune status of the respective dam [86,88]. Following recovery from clinical disease, some domestic ruminants can become reinfected with *E. ruminantium*, resulting in another bout of clinical disease or even death [47]. In addition, some animals may develop a transient carrier state. The mortality rate in clinically affected animals also varies considerably and is particularly dependent on when treatment is started during the course of the disease. Mortality rates vary between 5% and 10% [89]. 

The tropism of *E. ruminantium* for endothelial cells results in damage to capillaries. The resulting increased capillary permeability is particularly detrimental to cardiac and respiratory function. Primary clinicopathologic changes include progressive anemia, fluctuations in total and differential leukocyte counts (i.e., neutropenia, eosinopenia, and lymphocytosis), hyperbilirubinemia, hypoalbuminemia, decreased concentrations of total serum proteins, and azotemia. 

Infection of mice was the earliest method used to demonstrate variability between strains, and three different levels of pathogenicity are recorded: pathogenic genotypes which can kill mice, genotypes which infect mice but are not pathogenic, and non-infective genotypes. The closely related Panola Mountain *Ehrlichia* in the U.S. causes a mild febrile illness in goats and in humans [90,91], and its natural reservoir may be the widely distributed white-tailed deer [92]. Panola Mountain *Ehrlichia* has been found in *A. americanum* ticks, but does not appear to represent a heartwater threat [93]. 

There are important antigenic variants of *E. ruminantium*. For the purpose of vaccine development, it is important to find genotypes which can confer cross-immunity to as wide a range of strains as possible. The Welgevonden genotype is the only one which stimulates complete cross-protection against challenge with any of the other strains. 

Dogs with clinical manifestations suggesting canine ehrlichiosis had detectable *E. ruminantium*, suggesting that an *E. ruminantium* variant contributed to these animals’ illness [94]. Recently *E. ruminantium* DNA has also been detected in three human serum samples using the pCS20 assay. All three individuals died, and other *E. ruminantium*-specific gene sequences were also found in the samples, suggesting that at least some *E. ruminantium* variants can cause a lethal infection in humans [95].

## 6. Mouse Models of Ehrlichioses

While studies of ehrlichial infections in the natural hosts, such as *E. canis* in dogs and *E.* *ruminantium* in domestic ruminants, have defined the disease in terms of the pathologic lesions and pathophysiology as determined by the concentrations of biochemical analytes and cellular responses in blood, mechanistic studies of immunity and pathogenesis are most challenging in these outbred hosts. Descriptive studies, although essential, fail to determine the pathogenic and immune mechanisms. Such research is enabled by (1) the existence of susceptible and resistant inbred strains of animals that permit studies such as adoptive transfer of genetically compatible immune cell populations and (2) the availability of gene knockout animals lacking a specific gene or genes. Mice are the species in which such inbred susceptible, resistant, and gene knockout strains have been extensively developed. 

Of course, mice differ considerably from domestic ruminants, dogs, and humans. Yet they have been used most extensively to investigate ehrlichial immunity and pathogenesis. The challenge lies in interpreting the data from experiments in mice and applying the knowledge to veterinary and human clinical diseases.

Among the mice that have been used to study ehrlichial immunity, immunopathology, and pathogenesis are C57BL/6, C3H/HeN, C3H/HeJ, AKR, BALB/c, C.B 17, SCID, and knockout mice for genes CD4, CD8, MHC II, TAP, β_2_m, perforin, TNF receptor, IL-12, IL-21, and IL-18 receptors, TLR4, TLR9, TLR2, Rag, IFN-γ receptor, IFN-γ, IFN-α, TNF-α, lymphotoxin α, B cells (µMT), secretory IgM, class switching, and MyD88 [96,97,98,99,100,101,102,103,104,105,106,107,108,109,110,111,112,113,114,115,116,117]. These strains of mice have been utilized for studies of infections with species of *Ehrlichia* that cause subclinical self-limited infections, e.g., *E. chaffeensis* in C57BL/6 mice; mild-to-subclinical infections with lifelong persistence, e.g., *E. muris* in C57BL/6 mice; and severe, even uniformly, fatal infections, e.g., an unnamed *Ehrlichia* species obtained from Japanese *Ixodes ovatus* ticks (IOE). The route of inoculation affects the severity and outcome of infection, e.g., intraperitoneal infection with IOE is fatal except with the very lowest dose of *Ehrlichia*, and intradermal infection with IOE is sublethal except with the highest doses [118]. Infection of mice with the *E. muris* strain from the upper Midwestern United States illustrates these principles well. Intraperitoneal and intravenous inoculation with this *E. muris* strain causes dose-dependent lethality (higher dose) or sublethal persistent (lower dose) infection [119]. For as yet-to-be-determined reasons, mice that survive intravenous infection succumb to subsequent intravenous challenge, while mice surviving intradermal infection are immune to the same intravenous challenge dose. This strain is the only *Ehrlichia* that is pathogenic for humans for which a mouse model of ehrlichial acquisition by feeding of the natural vector, *I. scapularis*, on infected mice and transmission by infected ticks during feeding to uninfected mice has been developed [120]. In contrast with the intradermal needle inoculation that results in sublethal infection, tick transmission causes 27% lethality. The mechanisms mediating this enhanced severity of cutaneous inoculation by ticks are as yet undetermined. 

A mouse model that has played an important role in dissecting the mechanisms of immunity to lethal ehrlichial infection is challenge of mice that have been persistently infected with *E.* *muris* for 30 days or more with an ordinarily lethal dose of IOE [121]. These mice survive with strong *Ehrlichia-*specific secondary IFN-γ producing effector/effector memory CD4 and CD8 T cell responses, enhanced secondary anti-ehrlichial antibody response, accelerated bacterial clearance, and formation of granulomas.

## 7. Mechanisms of Pathogenesis and Immunity Determined in Experimental Mouse Studies

There are differences in some of the ehrlichial genes expressed in the tick and vertebrate hosts [122]. Expression of a greater number of genes occurs in ticks, and the course of infection in vertebrate hosts differs according to whether the source of ehrlichiae inoculated originated from infected tick cells or infected vertebrate cells in terms of the duration of infection and the humoral immune response [51,107,122].

Antibodies play an important role as a host defense against ehrlichiae. Mice lacking mature B cells that do not produce IgM or IgG, mice lacking Fcγ receptor I or complement receptors 1 and 2, or mice depleted of C3 die after inoculation with an ordinarily sublethal dose of IOE [123]. Although *E. muris*-primed wild type mice and mice genetically deficient in CD4 T cells, CD8 T cells, MHC class II, IL-12, IFN-γ, or TNF-α survive challenge with an ordinarily lethal dose of IOE, B cell-deficient mice succumb to the infection, indicating an important role for antibodies as a host defense in this model [108].

CD4 and CD8 T lymphocytes also contribute to immune control of ehrlichial infection. Although *E. muris* causes sublethal infection in wild type C3H/HeN mice, 81% of mice genetically deficient in MHC class I, 44% of CD4 T cell-depleted mice, and 80% of mice depleted of both CD4 and CD8 T cells die when infected with the same dose of *E. muris* [103]. Ordinarily, *E. chaffeensis* infection of mice is of short duration, but clearance is delayed in CD4 T cell knockout mice, and persistent infection occurs in MHC class II knockout mice [101,104]. A hint that CD8 T cells contribute to the pathogenesis of severe ehrlichiosis is the observation that 57% of CD8 T cell knockout mice survive, and the duration of survival is longer after infection with an ordinarily lethal inoculum of *E. ruminantium* [98]. The lethal IOE mouse model is characterized by overproduction of TNF-α by CD8 T cells, downregulation of IFN-γ production by CD4 T cells, low IL-12 in the spleen, and systemic overproduction of IL-10 [121]. Mice lacking a gene crucial for CD8 T cell function, β_2_ microglobulin, are highly resistant, and gene knockout mice for TAP (transporter associated with antigen processing) are substantially resistant to an ordinarily lethal dose of IOE in association with markedly lower TNF-α and IL-10 production, and restored CD4 T cell quantities including those producing IFN-γ. These data are consistent with CD8 T cells playing a role in excess production of TNF-α and IL-10 and the immune elimination of CD4 T cells [106]. Depletion of IL-10 restores the population of CD4 T cells in infected mice, indicating a role of this cytokine in the CD4 T cell depletion. A significant portion of the major histopathologic damage, hepatic injury, as well as control of bacterial infection is mediated by perforin. The pathogenic importance of TNF-α in IOE-induced liver injury is demonstrated by the greater resistance of mice deficient in both TNF receptors I and II (p55 and p75) [105]. TNF-α neutralization fails to increase resistance to IOE but may be less effective in blocking apoptotic signaling of the cell membrane form of TNF-α through TNF receptor p75. Infection of TNF receptor knockout and TNF-α neutralized mice results in a higher bacterial burden. Clearly, TNF-α plays both pathogenic and protective roles in IOE infection of mice. 

IL-18, which is highly expressed in the liver of mice during a lethal IOE infection, also contributes to the immunopathogenesis of fatal IOE infection. IL-18 receptor knockout mice have less host cell apoptosis, reduced hepatic inflammation, enhanced bacterial clearance, and prolonged survival compared with wild-type mice infected with IOE. Treatment of wild-type mice infected with mildly virulent *E. muris* with recombinant IL-18 increases hepatic injury and reduces bacterial clearance [115].

A contribution of natural killer (NK) cells to protective immunity against ehrlichiosis was demonstrated by the deaths of 80% of NK cell-depleted, *E. muris-*primed mice challenged with IOE [117]. In conclusion, various components of the immune system contribute to the pathogenesis of the disease process as well as to control of the infection. Three divergent immune-mediated outcomes of infection with different ehrlichial organisms are immune-mediated lethality associated with reduction in bacterial loads, subclinical persistence of infection, and transient sublethal infection.

Common clinical manifestations of ehrlichioses are thrombocytopenia, leukopenia, and anemia. Although there is evidence for mononuclear phagocytes removal of these elements, there are also investigations of the effects of ehrlichial infections of mice on the hematopoietic stem cells, hematopoietic progenitor cells, and myelopoiesis and the effects of innate and adaptive immunity on hemopoietic activity [109,110,111,124]. 

## 8. Prospects for Development of Vaccines

Vaccination ideally primes the immune system to develop memory cells that confront the invasive pathogen early in the incubation period to prevent the organism’s growth, dissemination, and disease mediation. Passive transfer of antibodies and adoptive transfer of immune cells of a specific subset can provide the host with individual immune components to evaluate their contribution to protective immunity that might be provided by vaccination. Passive transfer of not only polyclonal antibodies but also monoclonal antibodies, such as antibodies to the p28 expressed on *E. chaffeensis*, protect SCID mice against this agent [100,103]. Adoptive transfer of all three immune components stimulated by persistent *E. muris—*namely, polyclonal antibody and *Ehrlichia*-specific CD4 and CD8 T cells producing IFN-γ, each of which provides only partial protection individually—are required for strong protection against an ordinarily lethal IOE infection [121]. The role of *E. muris*-primed NK cells was demonstrated by protective immunity provided by their adoptive transfer into Rag2/interleukin-2 receptor knockout mice. These results suggest that an effective vaccine should stimulate the production of antibodies to specific proteins and memory CD4 and CD8 T cells and even memory-like NK cells that produce the appropriate cytokines including IFN-γ.

Much knowledge remains to be elucidated before rationally designed vaccines can be developed for human and veterinary ehrlichioses. A multi-passage attenuated strain of *E. canis* was experimentally tested as a vaccine against wild type bacteria, demonstrating protection in dogs [125]. Also, several vaccine trials have been performed for *E. ruminantium* [83,126]; however, immunization was not protective against *E. ruminantium* transmitted by natural tick challenge.

Because ehrlichiae are maintained in natural cycles for which no effective methods currently exist for control or eradication, vaccine development is the most compelling approach to ameliorate the effects of ehrlichiosis. A candid assessment is that greater attention to vaccine development is needed.
